# Cardiac Catheterization Post Congenital Cardiac Surgery: Analysis of Risk Factors for Mortality and Literature Review

**DOI:** 10.7759/cureus.67020

**Published:** 2024-08-16

**Authors:** Amin M Arfi, Jameel Alata, Haysam Baho, Zaheer Ahmad, Nashwa Badawy, Samia Bekheet, Wejdan Baatya, Abdelmonen Helal, Amjad Kouatli

**Affiliations:** 1 Pediatrics, King Faisal Specialist Hospital and Research Center, Jeddah, SAU; 2 Pediatric Cardiology, King Faisal Specialist Hospital and Research Center, Jeddah, SAU; 3 Pediatric Cardiology, Cairo University, Cairo, EGY

**Keywords:** pediatric cardiology, trans-catheter intervention, cardiac surgery, congenital heart defects, pediatric cardiac catheterization

## Abstract

Background

Diagnostic and interventional cardiac catheterization plays a significant role in the management of congenital heart defects with acceptable risks. Its role has also evolved in sick children but is associated with higher risks due to technical difficulties and co-morbidity factors. Some of the post-cardiac surgery children who show resistance to conventional management during the early postoperative period usually have residual defects or obstructions. Trans-catheter intervention (TCI) in such high-risk circumstances and relatively sick children is challenging, demands much expertise, and should be backed up by a competent multidisciplinary team. Some cases improve clinically, while others may require surgical or transcatheter re-intervention for a positive outcome. There is minimal data so far regarding the major complications after interventional cardiac catheterization during the immediate postoperative period after cardiac surgery. We analyzed multiple factors, including age, sex, weight, the initial diagnosis, and the time interval between surgery and TCI, to stratify the possible risks for mortality after TCI during the immediate postoperative period after cardiac surgery.

Results

Thirty-five patients fulfilled the inclusion criteria and underwent 43 interventional procedures. Five patients could not survive. Four had stent angioplasties on natural vasculature and one patient had in synthetic conduit. None of the mortality was related to the procedure. Multivariable risk factor analysis confirmed a moderate positive correlation coefficient (r) of 0.8017 between the variables. Still, it was not statistically significant if compared among subgroups or among the mortality and survival groups.

Conclusion

Interventional cardiac catheterization in sick children during the immediate postoperative period can be carried out without much-added risks in expert hands and under the supervision of a multi-disciplinary team. Though no conclusions could be drawn, our study adds to the limited existing data that could inspire others to perform such procedures on sick children. Moreover, the trend in our results indicated a large sample size could have identified a possible risk factor for mortality.

## Introduction

Diagnostic and interventional cardiac catheterization plays a significant role in managing congenital heart defects with acceptable risks. Over time, it has evolved into a therapeutic role [1] due to advances in device technology, interventional techniques, and an innovative approach to patient care [2]. All such procedures have been reported to be associated with some risks due to multiple factors, including age [3,4], weight [5,6], general condition of the patient [7], and the nature of the intervention [8,9]. Trans-catheter intervention (TCI) also has acquired a significant role during the early postoperative period in the management of children who had undergone surgical intervention for congenital heart defects. Some patients show improvement clinically while others might require additional surgical intervention, medical treatment, or mechanical circulatory support to treat these complications, such as a low cardiac output state. TCI during an early postoperative period is generally considered a high-risk procedure primarily due to the unstable clinical condition of children and, secondly, the finding of residual defects or obstructions after surgical repair. Multiple risk scoring systems exist for congenital cardiac surgery for minor risk stratifications, i.e., Risk Adjustment in Congenital Heart Surgery-1 (RACHS-1) score, Aristotle Basic Complexity (ABC) score, and Aristotle Comprehensive Complexity (ACC) score. However, minimal data has been published, regarding the major complications after interventional cardiac catheterization during the immediate postoperative period after cardiac surgery [10-12]. Our data, focusing on the efficacy and safety of such procedures under the same conditions has already been published [13]. In this retrospective analytical study, we have attempted to analyze multiple factors, including, age, sex, weight, the initial diagnosis, and the time interval between surgery and TCI, to stratify the possible risks for mortality after TCI during the immediate post-operative period after cardiac surgery.

## Materials and methods

Primary outcome

Major consequences, including mortality, were measured and examined for potential risk variables.

Study design

An open-label, observational retrospective cohort study.

Inclusions criteria

All patients who underwent cardiac surgery, off or on bypass, for congenital heart defects between January 2011 and December 2013 and who had significant issues, complications, or residual defects resulting in a prolonged stay in the Cardiac Surgical Intensive Care Unit (CSICU) and did not show improvement in clinical condition with conventional therapy, leading to non-discharge from CSICU and underwent interventional cardiac catheterization while still admitted in CSICU were included in the study. No limitations were imposed on the patients' minimum or maximum age or weight or on the minimum or maximum time interval between cardiac surgery and TCI for the inclusion or exclusion of the patients.

Exclusion criteria

Any patients who had been extubated and discharged from CSICU after cardiac surgery and all those who underwent diagnostic cardiac catheterization were excluded from the study.

Patient population

Over three years, 35 patients (21 males) fulfilled the criteria and were enrolled in the study. They underwent 43 procedures. The demographic details were recorded. The mean age of patients was 19.8 ± 38.3 months (0.3-192) and the mean weight was 7.4 ± 6.2 kg (1.9-33.9). The clinical data confirmed 15 patients having congenital heart defects with compromised pulmonary circulation (TOF/DORV with either pulmonary stenosis or atresia), nine patients had complex single ventricle morphology, and five patients had systemic arterial obstruction (coarctation). Two patients had d-TGA physiology, three patients had septal defects, and one had anomalous pulmonary venous drainage. The cardiac catheterization data were also reviewed. The time intervals, e.g., between cardiac surgery/TCI and between TCI/improvement in clinical status, were recorded. Procedure-related major complications such as mortality, were documented and subjected to statistical analysis for multivariable factorization, including age, weight, sex, the time interval between surgical intervention, and TCI.

Echocardiographic evaluation

All patients had an intraoperative transesophageal or epicardial echocardiographic evaluation before chest closure. Transthoracic echocardiography was also done in the CSICU as and when clinically indicated.

Cardiac catheterization

The decision for interventional procedure was made by a team comprising of, at least two pediatric cardiologists, a cardiac intensivist, and a cardiac surgeon. All procedures were done under general anesthesia in a biplane digital laboratory equipped for quick table-side quantitative analysis, image enhancement, and dynamic cine review at various speeds. The intensivist remained present in the same room throughout the procedure. Additionally, a standby team, including a pediatric cardiac surgeon, and a pediatric cardiac anesthetist, along with a ready-to-use operating room, was available. In critically sick children, a cardiopulmonary support unit was on standby. Standard techniques were applied for procedures, such as valvuloplasty, vascular/septal occlusion, and balloon/stent dilatation of vascular or synthetic conduits. Extra care was taken when crossing the newly created suture line by using high-torque floppy-tipped guide wires. 

Statistical analysis

The study recorded mortality in major diagnostic groups and in relation to various interventional procedures. Comparative analysis was conducted in two ways. Firstly, to analyze independent factors for mortality, five demographic and clinical variables, age, sex, weight, the time interval between surgery - TCI, and whether TCI was performed on native vasculature or synthetic conduits, were included. These independent factors were further divided into sub-groups in ascending order. For instance, age was divided into four subgroups: < 1 month, one to 12 months, 12-60 months, and > 60 months; weight was sub-grouped into < 2.5 kg, 2.6-5 kg, 5.1-10 kg, and > 10 kg; the time interval between surgery and TCI was divided into < 1 day, one to three days, three to five days, five to seven days and > 7 days. Statistical significance was analyzed using the Chi-square test to compare the categorical data of these factors and the results were presented in tabulated and graphical forms as applicable.

Secondly, the entire cohort was divided into survival and mortality. Multivariable analysis for risk factors among these two groups was conducted using the mean of the numeric data, applying the Spearman correlation analysis. The results were presented in figure and tabulated form.

## Results

The indications for TCI are presented in Table 1.

**Table 1:** Indications for trans-catheter intervention The data are presented in numbers and percentage form.

Indications	Number of patients	%age
Inability to wean off inotropic/ventilator support	30	61
Persistent cyanosis	7	14
Low cardiac output	5	10
Inability to wean off cardio-pulmonary support	5	10
Persistent effusions	2	4

**Figure 1:**
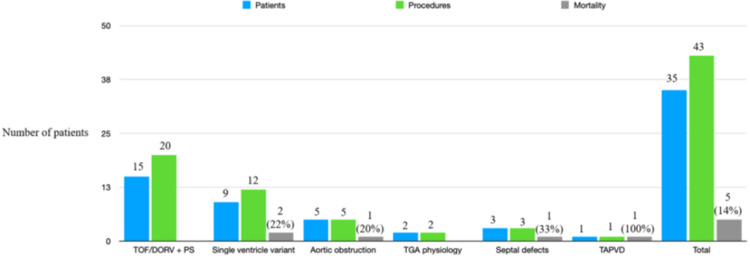
Mortality in different diagnostic groups Mortality in relation to different interventional procedures. The data are presented in numbers and percentage.

It can be seen that the major issue encountered by us was the inability to wean off either ventilatory support including failure of extubation or inotropic support. It is worth noting that five of the patients were on cardio-pulmonary support when they were taken for TCI. TCIs and mortality in major diagnostic groups are shown in Figure 1.

**Figure 2:**
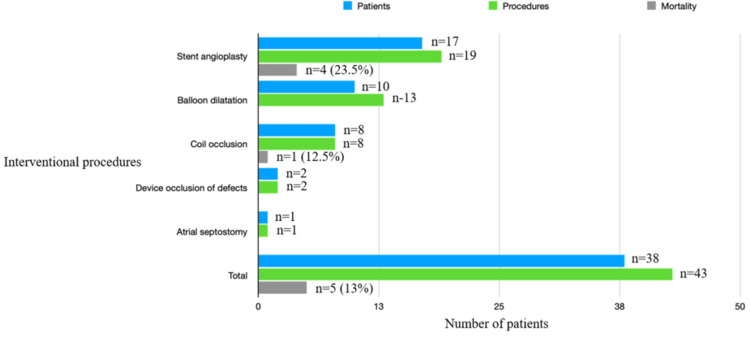
Mortality and interventional procedures Mortality in relation to interventional procedures. The data are presented in numbers and percentage Three patients had undergone more than one procedure and were counted in more than one group.

Application of the Wilcoxon matched pairs signed rank test indicated a significant difference between procedure and mortality. At the same time, the Spearman correlation analysis suggested a moderate positive correlation coefficient (r) of 0.8017 between the variables. Still, it was not statistically significant, indicating a larger sample size could have identified risk factors for mortality and diagnoses. Figure 2 shows the mortality among different interventional procedures.

A total of 43 procedures were performed on 35 patients. Five patients did not survive even after therapeutic cardiac catheterization. Among them, four had undergone stent angioplasty for significant residual obstruction on either native vasculature or on synthetic tubes/conduits. Three were on assisted ventilation, and two were on extracorporeal cardiopulmonary support. The cause of death in them was unrelated to the interventional procedures.

The comparison of mortality after TCI performed on natural vasculature versus synthetic conduits is presented in Tables 2, 3. Initially, the cohort was divided into two: natural vasculature (n=29) and synthetic conduit groups (n=6). A total of 37 procedures were performed on natural vasculature and six on synthetic conduits. Table 2 shows a correlation between the type of procedure at specific sites and mortality among the tested groups. Applying the Shapiro-Wilk test for normality confirmed the comparison was not statistically significant. Subsequently, dividing the cohort into three major procedural groups: stent angioplasties (n-18), balloon dilatations (n=14), and coil occlusions (n=9). A total of 35 procedures were performed on natural vasculatures and six procedures on synthetic conduits. Testing the mortality for each procedure done either on natural vasculature or synthetic conduits also confirmed that the comparison was not statistically significant (Table 3).

**Table 2:** Mortality in relation to natural vasculature versus synthetic conduits Correlation between type of procedure and mortality among the tested groups: natural vasculature versus synthetic conduits was conducted using the Shapiro-Wilk test for normality p<0.05 was considered significant. CoA: coarctation, PAs: pulmonary arteries, BTS: Blalock Taussig shunt, ASD: Atrial septal defect, MAPCAS: multiple aortopulmonary collaterals, PDA: patent ductus arteriosus, VSD: ventricular septal defect

Interventional procedures on natural vasculature (n=29)	Procedures	Patients (N)	Sites	Total numbers	Mortality	%age (mortality/total procedures x 100)	P-value
	Balloon dilatation	9	CoA=5	12	0	0	0.7950
			PAs=4				
			Glen=2				
			ASD=1				
	Stent angioplasty	12	PAs=7	15	4	27	
			Glen=3				
			CoA=2				
			Left bronchus=2				
			Carotid artery origin=1				
	Coil occlusion	6	MAPCAS=6	8	0	0	
			PDA=1				
			Venous=1				
	Device occlusion	2	VSD=2	2	0	0	
	Total	29		37	4	11	
Interventional procedures on synthetic conduits (n=6)	Balloon dilatation	2	BTS=2	2	0	0	>0.9999
	Stent angioplasty	3	BTS=3	3	0	0	
	Coil occlusion	1	BTS=1	1	1	100	
	Total	6		6	1	16	

**Table 3:** Comparison of mortality among natural vasculature versus synthetic conduits or tubes in relation to different procedures. Multivariable analysis for risk factors among the two groups: natural vasculature versus synthetic conduits, was conducted using the mean of the numeric data, applying the Chi-square test. p<0.05 was considered significant.

Procedures	Material	No. of patients	No. of procedures	No. of mortality	Percentage (%) (Mortality/procedures x 100)	P-value
Balloon dilatation	Native	9	12	0	0	0.387
	Synthetic	2	2	0	0	
Stent angioplasty	Native	12	15	4	26.7	0.290
	synthetic	3	3	0	0	
Coil occlusion	Native	6	8	0	0	0.125
	Synthetic	1	1	1	100	
Total	Native	27	35	4	11.4	0.805
	Synthetic	6	6	1	16.7	

Later, the cohort was tested for possible risk factors for mortality, including age (three sub-groups), weight (four sub-groups), sex (two sub-groups), the time interval between the surgery and TCI (five sub-groups). This multivariable risk factor stratification is provided in Table 4.

**Table 4:** Multivariable risk stratification for mortality Multivariable analysis for each risk factor for mortality among their sub-groups was conducted using the mean of the numeric data and applying the Spearman correlation analysis. p<0.05 was considered significant.

Factors	Groups	No. of patients	No. of procedures	No. of mortalities per patient	Percentage (%)	P-value
Age (months)	< 1	6	8	1	12.5	0.79
	1-12	18	21	3	14.3	
	> 12	11	14	1	7.1	
Weight (kg)	< 2.5	3	3	0	0	0.51
	2.6-5	12	14	3	21.4	
	5.1-10	12	15	1	6.7	
	> 10	8	11	1	9.1	
Sex	Male	21	27	3	11.1	0.89
	Female	14	16	2	12.5	
Time interval between surgery and intervention	< 1	3	3	0	0	0.69
	1-3	4	7	1	14.3	
	3-5	6	7	1	14.3	
	5-7	5	5	1	20	
	> 7	18	21	2	9.5	
TCI done on	Native vasculature	27	35	4	11.4	0.80
	Synthetic tube/material	6	6	1	16.7	

Finally, the mortality risk factors (age, weight, and the time interval between surgery and the TCI) were also tested among the mortality and survival groups. The comparison is shown in Table 5. Notably, none of the risk factors were statistically significant among mortality and survival groups.

**Table 5:** Multifactorial risk factor analysis Multivariable analysis for risk factors among the mortality and survival groups was conducted using the mean of the numeric data, applying the Spearman correlation analysis. p<0.05 was considered significant.

Factors	Group	Mean	SD	Range	P-value
Age (months)	Mortality	11	14.6	0.3-36	0.792
	Survival	21.2	40.9	0.3-192	
Weight (kg)	Mortality	6.6	6.3	2.7-17.5	0.513
	Survival	7.3	5.8	1.9-33.9	
Time * (days)	Mortality	7.8	5.4	3-17	0.399
	Survival	13.5	14.8	1-59	

It is quite evident from the results that none of the factors including the age, weight, sex, time interval between surgery and TCI, or nature of the procedure done, either on natural vasculature or on synthetic conduits contributed to the major complications in our cohort. The high standards of care could explain this in a potentially safe environment for the patients and may also be attributed to the expertise of our interventionists.

## Discussion

TCI has become a well-recognized and established part of managing congenital heart disease patients, serving as either the primary mode of treatment or a co-substitute before or after cardiac surgery. The safety and efficacy of TCI in older children and electively planned conditions have been extensively documented, demonstrating acceptable results and associated risks. However, specific interventional procedures in high-risk patients can carry a heightened risk of complications, including mortality [5,6,9]. Some of the risk factors for major complications have already been identified [3-9]. While TCI during the immediate post-operative period, after cardiac surgery, is logistically feasible, effective in outcome, and associated with a low risk of complications, there remains limited published data on this subject. In 2004, Zahn et al. [10] shared their experience with high-risk TCI in post-operative children after cardiac surgery, reporting no mortality in their series. In contrast, Ashosh et al. [14] reported a mortality of 43% in their 2009 experience involving 62 emergent cardiac catheterizations (35 interventions) in similar settings. In 2012, our study [13] was published, documenting TCI performed in sick children during the high-risk early postoperative period, demonstrating its safety and effectiveness with no procedure-related mortality. Later, in 2014, Nicholson et al. [11] conducted a comparative analysis of cardiac catheterization (diagnostic versus interventional) during the early postoperative period after cardiac surgery, concluding that there was no difference in survival to hospital discharge between the diagnostic and interventional groups (p=0.93). They also reported no variance in major or minor complications between the two groups. More recently, in 2018, Kasar et al. [12] published their experience of cardiac catheterization within 30 days after cardiac surgery in 50 children with intervention in 26 cases, including 16 children under extracorporeal membrane oxygenator support. Their series reported no procedure-related mortality. It is worth noting that the analysis of risk factors for mortality has rarely been scientifically stratified. In our current study, we aimed to identify factors associated with the risk of mortality after TCI performed in high-risk situations. While none of the tested risk factors were found to be scientifically significant in our study, our experience contributes to the ongoing exploration of safety and mortality after TCI in emergency or semi-emergency situations and sick children. Certain aspects of this discussion require further elaboration and consideration.

Complications associated with TCI in children

Since the inception of pediatric cardiac catheterization decades ago, there has been a consistent decline in reported complication rates, as noted by various authors. In 1992, Cassidy et al. [15] compared their contemporary experience with that of 20 years prior in pediatric cardiac catheterization. They demonstrated a substantial reduction in major complications, decreasing from 2.9% in the past to 0.9% in their current practice (p=0.0001). Additionally, they reported a decline in peri-catheterization mortality from 2.8% in the past to 0.2% in their present work (p=0.0001). This improvement in complication rates has been attributed to recent technological advances, enhanced imaging techniques, and the development of a diverse array of devices tailored specifically for use in children [16].

Several reports have aimed to analyze complications and associated risk factors in pediatric interventional cardiac catheterization. In 1998, Vitiello et al. [9] reported an overall complication rate of 8.8% in 4,952 consecutive cardiac catheterizations. Major complications accounted for approximately 2% of cases, with death occurring in 0.14% as a direct complication of the procedure. They concluded that younger age (up to two years) and interventional studies as compared to diagnostic cases, were major independent risk factors for morbidity and mortality.

TCI and the age/weight of children

Extensive research has been conducted to investigate the impact of cardiac catheterization on young and low-weight infants, often comparing them to their older counterparts. Rhodes et al. [5] conducted a comprehensive review in 2000, analyzing 2,042 cardiac catheterizations (1,685 weighing ≥ 5 kg and 357 ≤ 5 kg). Their findings indicated that a patient weight of ≤ 5 kg remained a significant risk factor for complications, regardless of the type of procedure performed. Specifically, within the weight range of 2.5-5 kg and ≤ 2.5 kg, a weight of ≤ 2.5 kg did not entail additional risks of complications. In 2001, Simpson et al. [6] shared their experience with 111 procedures in 107 infants weighing ≤ 2.5 kg, revealing a heightened risk of complications in interventional procedures compared to diagnostic catheterizations. More recently, Mory et al. [17] reported a major complication rate of 5.7% in children (1,558 procedures) compared to 2.3% in adults (576 procedures). Although our patient population comprised a relatively unwell group, we did not establish the age and/or the weight of the patients undergoing TCI as a risk factor for mortality in our series. A plausible explanation may lie in the early detection of issues, timely intervention, the presence of expert interventionists, and the high standard of post-operative ICU care.

TCI in sick children

Performing invasive interventions in post-operative children within the cardiac intensive care unit (ICU) setting is often met with hesitation, attributed to logistical challenges in patient transportation, infection control concerns, and the delicacy of fresh sutures [9]. It is reasonable to assume that the initial clinical condition of children undergoing TCI exposes them to a higher risk of complications, including mortality. However, this risk is likely associated with existing co-morbidity factors rather than the procedure itself. Children undergoing TCI during the immediate postoperative period typically exhibit compromised clinical conditions that necessitate immediate intervention. These children may present with refractory cyanosis, a low cardiac output state, or failed attempts at extubation. They are often confirmed to have residual anatomical defects and/or obstructive lesions, requiring urgent intervention to alleviate volume load, relieve pressure in a hypertensive ventricle, and maintain optimal oxygenation to improve cardiac output.

Several publications have reported favorable outcomes following TCI in high-risk settings. Zahn et al. [10] presented data on 66 catheterizations in 62 patients, including 50 interventions on 35 children during the immediate postoperative period. They reported a 100% success rate after angioplasty and vascular/septal occlusion, 87% after stent implantation, and 75% after palliative pulmonary valvotomy, with no procedural-related mortality. Their experience aligns closely with our results.

In 2009, Asoh et al. [14] shared the outcome of 62 emergent cardiac catheterizations (35 interventions) in 49 children in similar high-risk settings after pediatric cardiac surgery. They reported a high mortality of 43%, attributing delayed intervention beyond two to three weeks’ post-cardiac surgery and a splinted sternum as major risk factors for death. They concluded that invasive procedures during the immediate postoperative period and in the ICU settings were associated with poor outcomes, especially if delayed or impeded by multiple factors.

In 2012, Arfi et al. [13] published our initial experience with the efficacy and safety of TCI in sick children during high-risk early postoperative periods. Forty-three procedures were performed on 35 patients, demonstrating safety and effectiveness when conducted by expert hands and in a well-equipped catheterization laboratory supported by a multidisciplinary team.

In 2014, Nicholson et al. [11] shared their experience with 219 procedures (91 interventions) on 193 patients within 30 days of the postoperative period. They reported seven major complications, with one procedure-related mortality in the interventional group. Multivariate regression analysis identified a higher one-year mortality rate associated with young age at the time of surgery, ECMO support at the time of intervention, and single ventricle heart disease.

Our results align with some studies, while others report contrary findings. A plausible explanation may lie in the early identification and intervention of residual defects in our series, coupled with procedures conducted by expert hands and the support of a multidisciplinary team of sub-specialists.

Time interval between surgery and TCI

The reluctance to perform early intervention after cardiac surgery stems from concerns about the general critical condition of the patient, the vulnerability of the fresh suture line to stress, the risk of rupture during balloon dilatation or stent implantation, logistical challenges in transferring the patient from the pediatric intensive care unit to the cardiac catheterization laboratory, and potential breaches of infection control protocols. Our initial report [13] demonstrated that TCI under these high-risk settings is safe and effective, challenging the notion that early intervention poses additional risks.

Kentaro et al. [14] also reported that delayed intervention was a risk factor for mortality, emphasizing that early intervention after cardiac surgery was associated with better outcomes. They found that the fresh suture line was not an added risk factor for TCI after cardiac surgery. Our current results align with these findings confirming that the time between TCI and cardiac surgery was not an independent risk factor for mortality. This further supports the feasibility and safety of TCI during the early postoperative period, challenging traditional concerns about the timing of interventions in this context.

Limitations

Non-standardized Timing of TCI

The retrospective study introduced a limitation related to the non-standardized timing of TCIs. The timing was individualized based on clinical indications and the varied management approaches of multiple critical care attendees. This lack of standardization might affect the generalizability of the results.

Small Sample Size

Another limitation of the study is its small sample size, which restricts the generalizability of findings and the ability to make robust recommendations or suggestions. A larger sample size is essential for scientific validation and to establish standard recommendations for future clinical practice.

Selection Bias

The study's inclusion criteria, focusing on patients who failed to be discharged from the cardiac ICU and confirmed to have significant surgical residues, might introduce selection bias. This bias could impact the representativeness of the sample and, consequently, the applicability of the findings to a broader population.

Retrospective Analysis

The retrospective nature of the study design inherently comes with limitations such as reliance on historical data, potential incomplete records, and the inability to control for all variables. Prospective studies could provide more robust and controlled data.

Multifactorial Decision-Making

The decision-making process for TCI involved multiple critical care attendees, introducing the potential for varied management approaches and subjective clinical judgments. This multifactorial decision-making process may contribute to variability in the results.

Addressing these limitations through future research endeavors, such as prospective studies with larger sample sizes and standardized protocols, would enhance the scientific validity and generalizability of findings in pediatric cardiac catheterizations.

## Conclusions

Interventional pediatric cardiac catheterization can be carried out in electively planned settings and sick children during the immediate postoperative period without much-added risks, provided it is performed by expert hands and supported by a multidisciplinary team of specialists. While our study did not allow us to draw definitive conclusions regarding specific risk factors for mortality, an observed correlation coefficient (r) of 0.8017 was found between the number of procedures for diagnostic groups and the mortality, indicating a larger sample size could have identified risk factors for mortality to diagnoses. Moreover, the absence of identifiable risk factors still contributes valuable data. This information may encourage further exploration and implementation of such procedures for critically ill children in the early postoperative period, fostering positive implications for future clinical practice.
